# Imaging-driven diagnosis of retroperitoneal psoas major alveolar soft part sarcoma: a case report

**DOI:** 10.3389/fonc.2025.1609432

**Published:** 2025-11-25

**Authors:** Shasha Wei, Ming He, Wenfu Li, Lin Liu, Guang Ming Fan

**Affiliations:** 1 Department of Radiology, The Second Affiliated Hospital of Guizhou University of Chinese Medicine, Guiyang, Guizhou, China; 2 Department of Radiology, Guangdong Provincial People’s Hospital (Guangdong Academy of Medical Sciences), Southern Medical University, Guangzhou, Guangdong, China; 3 Department of Radiology, Affiliated Hospital of Zunyi Medical University, Medical Imaging Center of Guizhou Province, Zunyi, Guizhou, China; 4 Department of Radiology, Huizhou First People’s Hospital, Huizhou, Guangdong, China

**Keywords:** alveolar soft part sarcoma, retroperitoneal psoas major, imaging-guided diagnosis, multimodal imaging, rare tumor

## Abstract

Alveolar soft part sarcoma (ASPS) is an infrequent and malignant soft tissue tumor with an elusive tissue origin. Its occurrence in the retroperitoneal psoas major muscle is extremely rare. The tumor’s deep retroperitoneal location, complex anatomy, and hypervascularity pose challenges to preoperative diagnosis and surgical intervention. This case report presents a 21-year-old female diagnosed with left retroperitoneal psoas major ASPS. Advanced imaging modalities, such as computed tomography (CT) multidirectional reconstruction, magnetic resonance imaging (MRI), and three-dimensional (3D) rendering, were utilized. These imaging techniques not only clearly depicted the tumor’s characteristics but also its spatial relationships with surrounding tissues and the vascular network. The detailed preoperative vascular assessment enabled the surgical team to comprehend the tumor anatomy and meticulously plan the approach, thus significantly reducing surgical risks and potential complications. The procedures and outcomes of this case offer valuable insights for clinical practice, highlighting the crucial role of imaging in the diagnosis and treatment of rare ASPS cases.

## Introduction

Alveolar soft part sarcoma (ASPS) is a rare malignant soft tissue, predominantly affecting adolescent female individuals (1, 2). It often presents with subtle clinical manifestations, which may lead to delayed diagnosis. ASPS is highly vascular and metastatic, necessitating early diagnosis and prompt intervention to improve survival and mitigate recurrence risks. In the present case, the involvement of the retroperitoneal psoas major muscle represents a distinct and rare anatomical site for ASPS, with only two reported cases identified in the English literature based on our review (3, 4). The unique anatomical location of the psoas major muscle, deep within the retroperitoneum, surrounded by vital organs, major blood vessels, and nerves, complicates the diagnosis and treatment. Due to the tumor’s proximity to critical structures such as major vessels, preoperative advanced imaging was essential for comprehensive evaluation and surgical planning. This case highlights the significance of detailed imaging evaluation in accurately diagnosing ASPS and planning effective surgical interventions, especially in rare anatomical locations.

## Case presentation

A 21-year-old female presented with a six-month history of recurrent mild left lower abdominal pain. Initially intermittent (2–3 episodes weekly), the pain progressed to daily occurrences over the past two months, with moderate intensity. Physical examination revealed mild tenderness localized to the left lower quadrant. On physical examination, mild tenderness was detected in the affected area.

**Figure 1:**
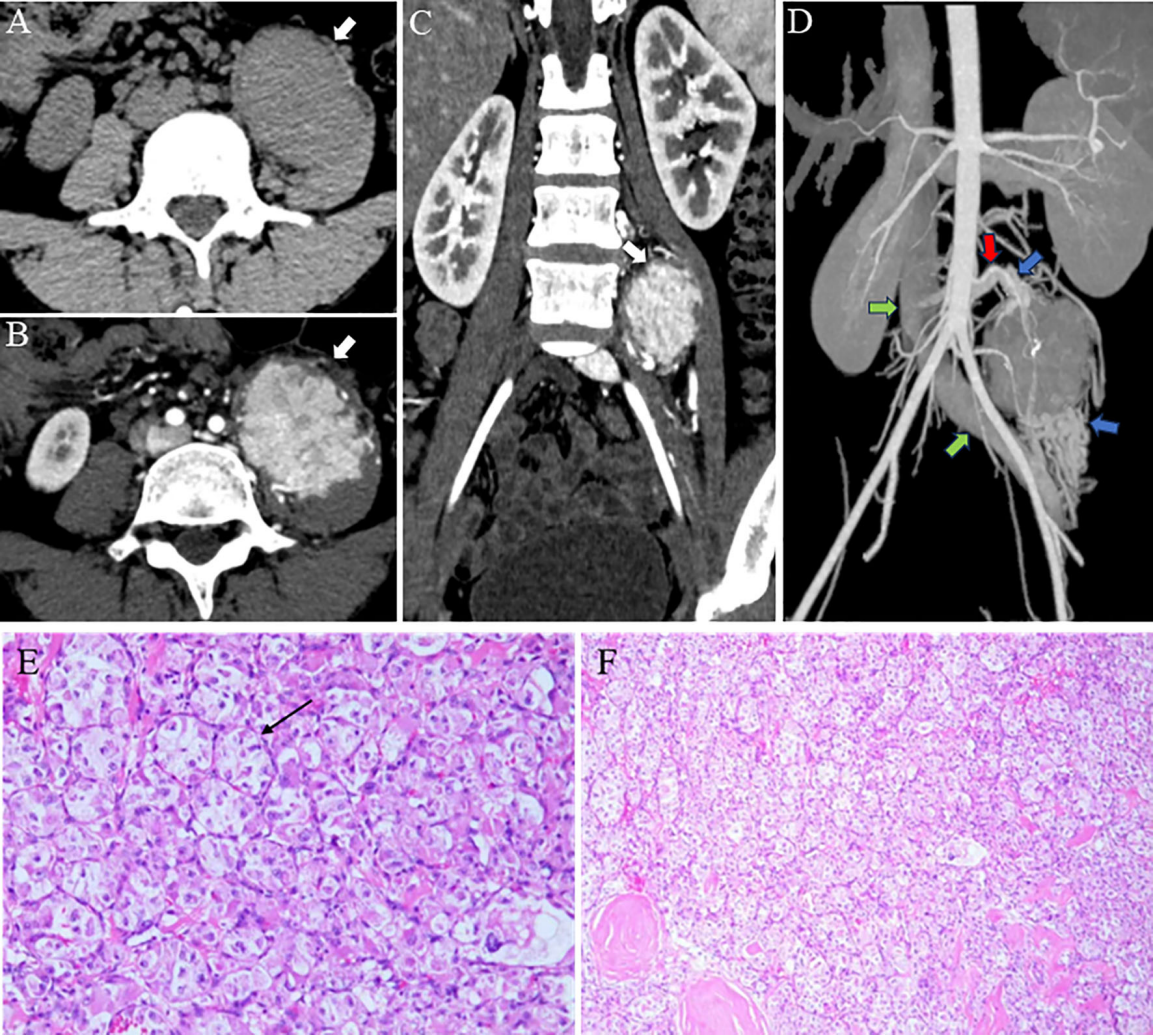
Imaging findings. **(A)** A clearly defined, isodensed mass located within the left retroperitoneal psoas major muscle is revealed in CT. **(B)** A highly vascular tumor with internal nodularity and lobulated margins (white arrow) is seen on a contrast-enhanced CT axial image. **(C)** A reconstructed coronal image displaying the “split-fat” -a radiological term describing fusiform fat-density shadows encircling the tumor’s upper and lower poles, sharply contrasting with its soft-tissue density, reflecting fat displacement caused by tumor expansion (white arrow). **(D)** A maximum intensity projection image showing large peripheral vessels located at the superior and inferior regions of the tumor, revealing multiple branch supply arteries originating from the abdominal aorta (red arrow), along with tortuous and thickened veins surrounding the mass (blue arrow), which ultimately drains into the inferior vena cava (green arrow). **(E, F)** Tumor cells are organized in an alveolus-like structure, with delicate blood sinuses present in the interstitium (black arrow).

The patient’s initial symptom was left lower abdominal pain. Laboratory findings were unremarkable. Therefore, a rapid, non-contrast abdominal CT scan was performed to rule out common causes of acute abdominal pain, such as urinary calculi. Non - contrast CT showed a well - defined, isodense mass within the left retroperitoneal psoas major muscle (**Figure 1A**). The patient’s serum creatinine and blood urea nitrogen levels were within normal limits, indicating no significant renal impairment; therefore, contrast-enhanced CT was performed, revealing a hypervascular tumor lesion. The tumor presented as nodular aggregates with distinct lobulated margins (**Figure 1B**). It was located in the interstitial space of the psoas major muscle, and a splitting sign of the muscle was visible on coronal views (**Figure 1C**). The upper and lower poles of the tumor had a complex network of thick and tortuous vessels. These vessels were supplied by multiple arteries branching from the abdominal aorta and drained into the inferior vena cava and the left iliac vein (**Figure 1D**). The Three-dimensional (3D) cinematic rendering clearly depicted the tumor nodule aggregates arranged in a glandular vesicle - like pattern, along with the elaborate vascular network around the tumor, including the feeding arteries and draining veins.

**Figure 2:**
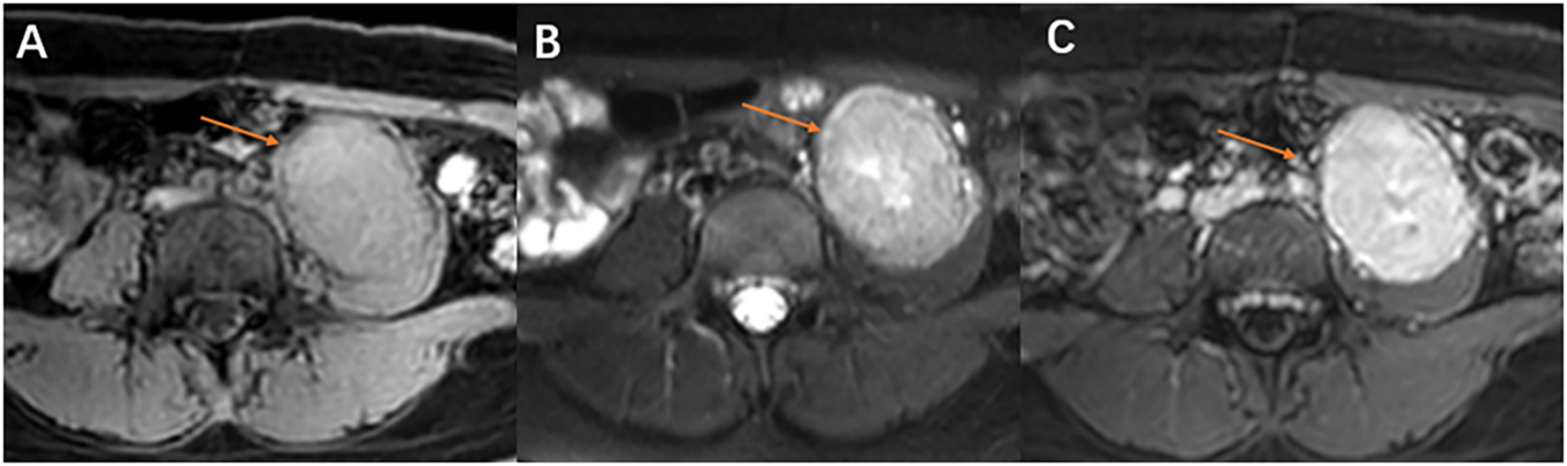
MRI, Magnetic resonance imaging reveals the tumor in the left psoas major muscle (red arrow). **(A)** On T1WI, the mass in the left psoas major, with a round-like appearance, demonstrated an isosignal. **(B)** On T2WI, the mass exhibited a high signal, with a slightly elevated signal visible in the center. Tiny blood vessels are visible at the edge. **(C)** The MRI enhancement scan indicates a notable blood-rich enhancement in the tumor of the left psoas major muscle.

MRI Findings: Magnetic resonance imaging (MRI) was performed. On T1 - weighted images (T1WI), the tumor showed an isointense signal (**Figure 2A**). On T2 - weighted images (T2WI), it exhibited a slightly hyperintense signal (**Figure 2B**). After contrast administration, there was significant enhancement, suggesting rich blood supply (**Figure 2C**).

Surgical and Gross Findings: Due to preoperative imaging findings indicative of a highly malignant, hypervascular tumor, the patient underwent radical tumor resection. During the operation, it was found that the tumor had poor demarcation from the adjacent muscles and limited mobility. The psoas major muscle was dissected to facilitate the resection. Grossly, the tumor was grayish - white, with focal gelatinous areas, multiple hemorrhagic foci, and a medium - textured surface.

Microscopic and Molecular Findings: Microscopically, the tumor cells had ovoid nuclei and prominent nucleoli. The cytoplasm was slightly basophilic. The cells were arranged in solid nests with focal hemorrhages, and the stroma contained sinusoidal blood vessels (**Figure 1E, F**). Molecular pathology testing using fluorescence *in situ* hybridization (FISH) revealed positivity for TFE3. Immunohistochemical analysis also showed strong positivity for TFE3 (++++).

Diagnosis and Follow - up: Based on the imaging features, pathological characteristics, and immunohistochemistry results, a final diagnosis of alveolar soft part sarcoma was made. The patient underwent postoperative surveillance with contrast-enhanced abdominal CT or MRI at 6 and 12 months. By 18 months, all imaging studies showed no evidence of local recurrence or distant metastasis.

## Discussion

ASPS is a rare, highly vascularized malignancy of soft tissue (5), accounting for less than 1% of all soft tissue tumors (6, 7). Predominantly affects adolescents and young adults, with a slight female predominance (8). ASPS can occur in a wide range of locations (9). In adults, it most commonly involves the deep muscles of the limbs and trunk, with the thighs being affected in up to 40% of cases (10, 11). In children, ASPS tends to develop in the head and neck region (12, 13) and is characterized by a high metastasis rate and recurring tendency. It also poses a significant risk of bleeding during biopsy. Metastasis primarily occurs in the lungs, bones, and brain (14). Approximately 50–70% of patients with metastases at diagnosis or during progression (15, 16). Due to its rarity and subtle early symptoms, which often manifest as painless lumps or mild pain, clinicians have limited familiarity with this disease, often leading to misdiagnosis, missed diagnosis, or delayed treatment.

The histological origin of ASPS remains undetermined. Microscopically, ASPS manifests unique and striking morphological features. The most notable features involve the aggregates of vesicular or organoid structures, which are among the hallmark features of this tumor and important for its pathological diagnosis (16). Tumor cells are separated by fibrovascular interstitium, forming nests or vesicular structures of different sizes, surrounded by thin-walled blood vessels (17). These features are crucial for pathological diagnosis. At the molecular level, the hallmark alteration of ASPS is the ASPSCR1-TFE3 gene translocation and the consequent formation of its fusion gene. This fusion gene encodes a chimeric protein that functions as an aberrant transcription factor, promoting tumor angiogenesis and cellular proliferation by activating the MET signalling pathway. In diagnostic practice, while TFE3 immunohistochemistry demonstrates high sensitivity, its specificity is limited; therefore, detection of ASPSCR1-TFE3 fusion transcripts is regarded as the definitive diagnostic criterion for ASPS (9, 12).

On CT, the tumor was visualized as a well-defined round-like soft tissue mass with expansive growth characteristics, consistent with previously reported cases (18). On enhanced CT, the tumor showed nodular aggregates with markedly homogeneous enhancement and lobulated margins, with a significantly higher degree of enhancement than that of the surrounding muscle tissue. This enhancement pattern is one of the important features of ASPS (8). In addition, thick and tortuous blood supply and drainage vessels can be seen at the tumor edge; this vascular distribution pattern is related to the growth supply of the tumor. It has been suggested in the literature that if lesion is combined with metastases, it is suggestive of ASPS (19). These imaging features provide valuable information for informing clinicians to make tumor diagnosis and avoid surgical hemorrhage during surgery.

CT multidirectional reconstruction imaging enables observation of tumor and its adjacent foci from various perspectives. In the present case, the coronal image revealed that the tumor had penetrated the psoas major muscle, manifesting the “fat split sign” (This imaging finding represents expansile growth of the tumor within the intermuscular septa, displacing and splaying apart the surrounding muscle bundles, thereby rendering the intermuscular fat planes more conspicuous.) (20). 3D rendering imaging technology clearly and intuitively demonstrated the tumor’s morphology, with the clusters and arrangements of nodules resembling adenoids, as well as the intricate network of blood vessels encircling the tumor.Study have revealed a characteristic distribution of a deep tumor with over 5 thick blood vessels located in the center or surrounding the tumor, primarily in the upper and lower poles of it. This feature could potentially be indicative of ASPS (21). Furthermore, 3D rendering non-invasively delineated the anatomical relationship between the tumor and adjacent key vasculature. This provided critical guidance for formulating a meticulous surgical plan, enabling the team to preoperatively define the surgical approach, identify critical vascular structures, and develop strategies to preserve them, with the aim of reducing the risk of intraoperative haemorrhage.MRI provided additional insights into ASPS diagnosis. The tumor exhibited an isosignal on T1WI and a marginally brighter signal on T2WI. Within the tumor, there were small patches with a brighter signal, likely indicating potential areas of liquefied necrosis. It has been proposed that the high signal detected in certain blood sinuses located at the tumor center could play a crucial role in ASPS diagnosing (21).

The imaging findings of this study closely align with the features summarised in a recent systematic review, which integrated single-institution experience with all published ASPS imaging research. The main imaging hallmarks of ASPS outlined in the literature include deep location, slightly hyperintense signal on T1WI, hyperintense signal on T2WI, abundant flow voids, marked intratumoural vascularity, and prominent peritumoural feeding vessels (22).

In this case, the tumor was located in the deep retroperitoneum. The MRI manifestations included an isointense signal on T1WI (with signal intensity comparable to muscle but higher than water), slightly hyperintense signal on T2WI, and markedly hypervascular enhancement following contrast administration. Particularly noteworthy was the presence of fine vascular flow voids surrounding the tumor on T2WI, corresponding to the “abundant flow voids” described in the literature. Contrast-enhanced CT further confirmed the existence of substantial peritumoural feeding vessels.

Although this case demonstrated a slight deviation from the typical presentation in T1WI signal intensity, its deep location, T2WI signal characteristics, vascular flow voids, marked enhancement pattern, and characteristic vascular architecture collectively exemplify the typical imaging features of ASPS (18).

In summary, CT, MRI, and 3D rendering serve complementary roles in the imaging diagnosis of ASPS, with their respective characteristics compared in **Table 1**.

**Table 1:** Comparison of advantages and limitations of major imaging modalities in alveolar soft part sarcoma (ASPS).

Imaging modality	Major advantages	Major limitations
Non-contrast CT	Rapid examination; sensitive for detecting calcification, haemorrhage, and high-density calculi; suitable for emergency screening.	Poor soft tissue contrast; unable to assess tumour vascularity.
Contrast-enhanced CT	Clearly demonstrates marked, homogeneous tumor enhancement; effectively visualises coarse, tortuous feeding arteries and draining veins; excellent for evaluating bone invasion.	Involves ionising radiation and risk of iodine contrast allergy; inferior to MRI in resolving fine soft tissue details.
MRI	Excellent soft tissue resolution for defining anatomical relationships with muscles and nerves; multi-parameter imaging provides characteristic signal information	Longer examination time; insensitive to calcification.
3D Rendering	Intuitive, three-dimensional display of tumor morphology, nodular clustering (alveolar-like pattern), and 3D vascular network; greatly aids surgical planning and doctor-patient communication.	Image quality is highly dependent on the quality of the original scan data.

Retroperitoneal alveolar soft part sarcoma (ASPS) requires differentiation from the following entities. Rhabdomyosarcoma (RMS): Primarily affects children and adolescents. While both ASPS and RMS exhibit iso- to hypointense signals on T1WI (23), RMS is characterized by rapid growth, high-grade malignancy, intratumoral hemorrhage/necrosis, and heterogeneous enhancement—distinct from ASPS’s homogeneous enhancement and nodular alveolar-like architecture (24). Paraganglioma (PGL): A common retroperitoneal tumor, often presenting as a well-defined oval mass with marked contrast enhancement, mimicking ASPS (25). Key distinguishing features of PGL include intratumoral necrosis, cystic changes, and tortuous vascular shadows, which are rare in ASPS (26).

Complete surgical resection has traditionally been regarded as the optimal treatment of ASPS (27). Furthermore, for metastatic or refractory cases, combined targeted therapy and immunotherapy show promise (9, 16, 28). The choice of treatment should be individualized based on the tumor’s location, size, stage, and the patient’s overall condition. In this case, the successful surgical resection, aided by detailed preoperative imaging, contributed to the favorable short-term outcome. Given the significant propensity of alveolar soft part sarcoma (ASPS) for late recurrence and metastasis, with post-resection survival rates declining from 95% at 1 year to 86% at 2 years and 73% at 5 years (16). we strongly recommend implementing a long-term (≥5 years) systematic follow-up protocol. This should include regular imaging surveillance [CT, MRI, or PET-CT(Positron Emission Tomography-Computed Tomography)] at 6- to 12-month intervals to facilitate early detection of disease progression. If recurrence or metastasis occurs, prompt initiation of comprehensive management strategies incorporating targeted therapy and immunotherapy may improve long-term survival outcomes (9).

## Summary and limitations

This case report is subject to inherent limitations, including its single-centre nature, its focus on an individual case of a rare condition (with only two cases reported to date; imaging features summarised in **Table 2**), and its lack of a control group, which restrict the generalizability of the findings. However, it is worth emphasizing that the imaging characteristics observed in this case show strong consistency with those documented in a systematic review, thereby lending support to their representativeness. Furthermore, the MRI evaluation in this study was primarily based on conventional sequences and did not include quantitative analysis of Diffusion-Weighted Imaging (DWI) or Apparent Diffusion Coefficient (ADC). Existing research indicates that these functional parameters can provide additional information on tumor biology and improve the overall accuracy of imaging diagnosis for ASPS (8).

**Table 2:** Summary of imaging features in reported cases of psoas major ASPS.

Reference	Cases	Imaging features
2010	1	MRI: T1WI iso-/slightly hyperintense (relative to muscle), T2WI hyperintense; markedly hypervascular enhancement CT: Hypervascular enhancement with central low-density necrosis on contrast-enhanced scans Angiography: demonstrated a close relationship with lumbar artery and left internal iliac artery
2017	1	CT: Hypervascular enhancement with internal low-density necrosis on contrast-enhanced scans
Present Case	1	MRI: T1WI isointense, T2WI slightly hyperintense; markedly hypervascular enhancement; vascular flow voids visible CT: Uniform hypervascular enhancement on contrast-enhanced scans; coarse feeding vessels visible 3D rendering: Clearly demonstrates “alveolar-like” nodular clusters and 3D vascular network

In conclusion, this case thoroughly characterises the presentation of ASPS in the rare psoas major location through multimodal imaging incorporating CT, MRI and three-dimensional rendering. The 3D rendering vividly demonstrates the tumor’s morphological features and its anatomical relationship with major blood vessels, thereby providing crucial anatomical guidance for surgical planning, intraoperative haemorrhage risk assessment and complete tumor resection.

## Data Availability

The original contributions presented in the study are included in the article/**Supplementary Material**. Further inquiries can be directed to the corresponding author.

## Supplementary material

Supplementary Video. Three-dimensional rotating movie of the cinematic rendering of alveolar soft part sarcoma in the left psoas major.
